# Cancer Risk in IBD Patients Treated with JAK Inhibitors: Reassuring Evidence from Trials and Real-World Data

**DOI:** 10.3390/cancers17050735

**Published:** 2025-02-21

**Authors:** Pierluigi Puca, Angelo Del Gaudio, Jacopo Iaccarino, Valentina Blasi, Gaetano Coppola, Lucrezia Laterza, Loris Riccardo Lopetuso, Stefania Colantuono, Antonio Gasbarrini, Franco Scaldaferri, Alfredo Papa

**Affiliations:** 1 Department of Medical and Surgical Sciences, Catholic University of the Sacred Heart, 00168 Rome, Italy; pierluigi.puca@unicatt.it (P.P.); angelo.delgaudio01@icatt.it (A.D.G.); jacopo.iaccarino01@icatt.it (J.I.); valentina.blasi01@icatt.it (V.B.); lorisriccardo.lopetuso@guest.policlinicogemelli.it (L.R.L.); antonio.gasbarrini@unicatt.it (A.G.); franco.scaldaferri@unicatt.it (F.S.); 2 IBD Unit, Digestive Diseases Centre (CEMAD), Department of Medical and Surgical Sciences, Policlinico Universitario “A. Gemelli” Foundation, IRCCS, 00168 Rome, Italy; gaetano.coppola@guest.policlinicogemelli.it (G.C.); lucrezia.laterza@policlinicogemelli.it (L.L.); stefania.colantuono@policlinicogemelli.it (S.C.)

**Keywords:** JAK inhibitors, malignancies, cancer, tofacitinib, upadacitinib, filgotinib, inflammatory bowel disease

## Abstract

The advent of Janus kinase (JAK) inhibitors, including tofacitinib, filgotinib, and upadacitinib, has significantly widened the therapeutic options for patients with inflammatory bowel disease (IBD). These agents offer the advantage of oral administration and have demonstrated efficacy in inducing and maintaining remission. However, concerns regarding their safety have emerged, particularly concerning cardiovascular and infectious complications, which appear more pronounced in patients with pre-existing risk factors such as older age, smoking, or comorbidities. While these risks are better understood, the potential association between JAK inhibitors and malignancies remains a subject of ongoing investigation. Current data from randomised controlled trials, pooled and integrated analyses, and real-world studies provide conflicting evidence regarding cancer risk. Notably, studies in patients with rheumatologic diseases treated with JAK inhibitors have contributed additional insights into long-term safety outcomes. Despite the uncertainty surrounding malignancy risks, it is likely that predisposing factors, including older age, smoking history, and long-standing IBD with chronic inflammation, play a more substantial role in cancer development than JAK inhibitor therapy alone. This paper reviews safety data from clinical trials, meta-analyses, and observational studies, focusing on cancer risk in patients treated with JAK inhibitors for IBD. We also review evidence from rheumatology studies, highlighting the need for individualised risk assessment and close monitoring to optimise the safety profile of these medications in clinical practice.

## Simple Summary

Janus kinase inhibitors are effective oral therapies for inflammatory bowel disease. While these drugs improve treatment options, safety concerns remain, particularly regarding cardiovascular and infectious risks, especially in patients with existing risk factors. The relationship between these medications and cancer risk is less clear, with conflicting evidence from clinical trials and real-world studies. Factors like older age, smoking, and long-standing IBD may influence cancer risk more than the medications themselves. This review summarises current safety data, emphasizing the need for careful patient selection, individualised risk assessment, and monitoring during treatment.

## 1. Introduction

Inflammatory bowel disease (IBD), encompassing conditions such as Crohn’s disease (CD) and ulcerative colitis (UC), poses significant challenges in management due to its chronic nature and potential complications. The advent of Janus kinase (JAK) inhibitors has widened the treatment landscape for IBD, offering new options for therapeutic intervention [1].

### 1.1. The JAK/STAT Pathway

JAK inhibitors function by selectively blocking the activity of one or more of the JAK family of enzymes (JAK1, JAK2, JAK3, and Tyk2). The JAK-signal transducers and activators of transcription (STAT) pathway is pivotal in various immune processes, including hematopoiesis (the formation of blood cells), immune cell development (such as T cells and B cells), and inflammatory responses. Dysregulation of this pathway can lead to immune disorders or contribute to cancer development [2].

The JAK–STAT pathway is a vital signalling mechanism that mediates cellular responses to various extracellular signals, particularly cytokines and growth factors. This pathway regulates immune responses, cell growth, differentiation, and survival. The JAK–STAT pathway consists of three main components: cytokine receptors, Janus kinases, and signal transducers and activators of transcription (STATs). When a cytokine binds to its specific receptor on the cell membrane, it induces a conformational change that brings associated JAKs into proximity. This proximity activates the JAKs through a process called trans-phosphorylation. Once activated, JAKs phosphorylate specific tyrosine residues on the receptor, creating docking sites for STAT proteins. The binding of STATs to these phosphorylated residues allows JAKs to phosphorylate the STAT proteins, leading to their activation. The activated STATs then dimerise as homodimers or heterodimers and translocate to the nucleus. In the nucleus, they bind to DNA and regulate gene transcription related to immune responses, cell growth, and differentiation [3,4].

**Figure 1:**
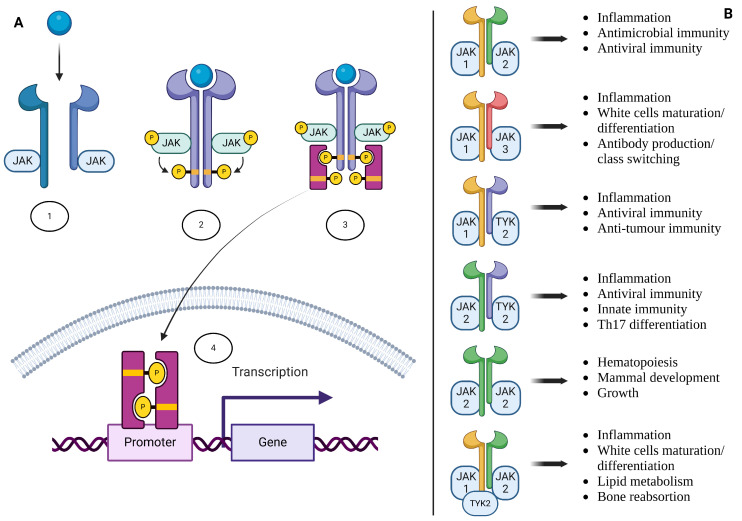
JAK-signal transduction pathway and its role in cellular signalling and immune regulation. (**A**) The schematic representation shows the JAK–STAT signalling cascade: (1) Cytokine binding induces receptor dimerisation, activating JAKs. (2) JAKs phosphorylate the receptor and recruit STAT proteins. (3) Phosphorylated STATs dimerise and translocate to the nucleus. (4) STATs bind DNA promoters, initiating gene transcription. (**B**) Different JAK pairings and their downstream effects, highlighting specific immune and inflammatory processes such as antiviral immunity, antibody production, Th17 differentiation, hematopoiesis, and bone metabolism. This pathway is a key target for therapies addressing inflammatory and autoimmune diseases.

**Figure 2:**
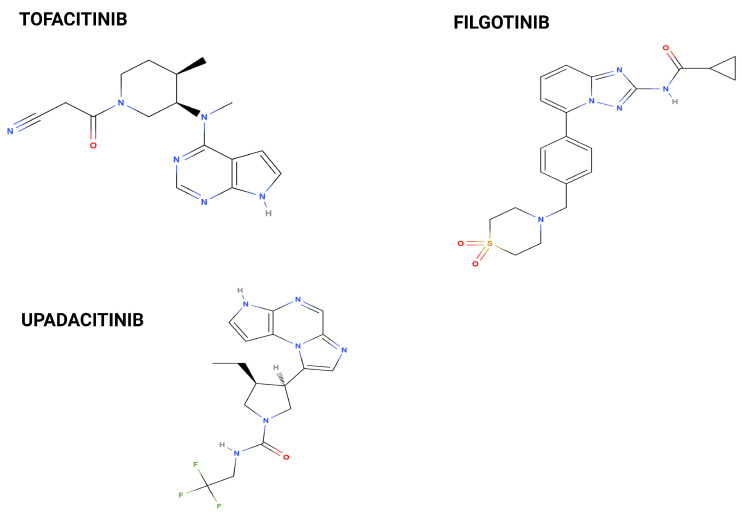
Molecular structure of tofacitinib, filgotinib, and upadacitinib.

There are four primary isoforms of JAK in mammals: JAK1, JAK2, JAK3, and TYK2. Each isoform has distinct functions and tissue distributions that allow specific signalling outcomes depending on the cytokines involved. JAK1 is ubiquitously expressed across various tissues and is primarily involved in signalling for type I interferons and several interleukins, such as IL-6. It plays a crucial role in both innate and adaptive immunity. JAK2 is also widely expressed but is particularly prominent in hematopoietic cells. It is critical for signalling through receptors for erythropoietin (EPO) and thrombopoietin (TPO), which influence blood cell production. Mutations in JAK2 have been associated with myeloproliferative disorders. JAK3 is primarily expressed in hematopoietic cells and is specifically involved in signalling through common gamma chain cytokine receptors, which include IL-2 and IL-4. This isoform is essential for T cell development and function; its deficiency can lead to severe combined immunodeficiency. TYK2 is expressed in various tissues but is less ubiquitous than JAK1 and JAK2. It is involved in signalling for type I interferons and IL-12, playing a role in antiviral responses. TYK2 has also been implicated in autoimmune diseases [5]. Figure 1 summarises the functioning of the JAK/STAT pathway and the downstream effect of different isoforms’ dimerisation. Figure 2 depicts the molecular structure of the three JAK inhibitors approved for IBD.

### 1.2. JAK Inhibitors for IBD: Effectiveness and Safety

Three JAK inhibitors have been approved for the therapeutic armamentarium of IBD. The first JAK inhibitor approved for UC was tofacitinib. Subsequently, filgotinib was approved for UC, and upadacitinib was approved for both UC and CD. Different JAK inhibitors have varying selectiveness for JAK isoforms. Tofacitinib primarily inhibits JAK1 and JAK3, while upadacitinib and filgotinib are selective for JAK1 [6].

A first-generation JAK inhibitor, tofacitinib presents the highest affinity for JAK1 and JAK3, followed by JAK2. After phase II and III clinical trials, it was approved for the treatment of UC in 2018. A multi-centre cohort study (REMIT-UC) enrolled 334 adult outpatients with UC and evaluated response to tofacitinib up to 52 weeks. In total, 35.3%, 36.0%, and 35.2% of patients achieved clinical response after 12.24 and 52 weeks, respectively. Parallelly, 18.5%, 23.0%, and 25.7% achieved an endoscopic response, showing that tofacitinib is effective in inducing and maintaining clinical response in UC [7].

A second-generation JAK1 inhibitor, filgotinib, has been approved to treat UC. The Phase IIb/III SELECTION trial evaluated the effectiveness of this medication in inducing and maintaining response UC. Patients receiving filgotinib 200 mg QD showed a higher clinical response rate than those receiving filgotinib 100 mg QD or placebo at week 10. However, when assessing the clinical response rates at week 58, the clinical response rate for patients receiving filgotinib 100 mg QD was significantly higher than that in the placebo group [8].

Finally, the JAK1 selective upadacitinib was the first JAK inhibitor to be launched for both UC and CD. Key findings from the phase II CELEST trial indicated that upadacitinib effectively induced clinical remission and showed significant improvements in endoscopic outcomes in patients with CD having failed anti-tumour necrosis factor (TNF)-α [9]. Such trends were confirmed in phase III induction and maintenance trials (U-EXCEL and U-EXCEED). In U-EXCEL, 49.5% of patients achieved clinical remission compared to 29.1% on placebo, while in U-EXCEED, the figures were 38.9% versus 21.1%, with endoscopic responses of 46% and 34.6%, respectively. The U-ENDURE study aimed to evaluate the effectiveness of upadacitinib in maintaining remission in CD. Five hundred-two patients were randomly selected to receive 15 or 30 mg of upadacitinib or a placebo for 52 weeks. The results showed that significantly more patients treated with upadacitinib achieved clinical remission at week 52 compared to placebo, with rates of 37.3% for the 15 mg group and 47.6% for the 30 mg group versus 15.1% for placebo [10]. The effectiveness of upadacitinib in UC has been proven by the U-ACHIEVE and U-ACCOMPLISH trials. The U-ACHIEVE and U-ACCOMPLISH trials demonstrated that upadacitinib (45 mg once daily) effectively induces clinical remission in patients with moderate to severe ulcerative colitis, with U-ACHIEVE showing 33% of patients achieving remission at week 8 compared to 4% on placebo, and U-ACCOMPLISH reporting a clinical response rate of 74% versus 25% for placebo. Both trials met their primary endpoints, with significant improvements in endoscopic outcomes; U-ACHIEVE reported 44% of patients achieving endoscopic improvement compared to 8% on placebo. The U-ACHIEVE maintenance study demonstrated that upadacitinib effectively maintains clinical remission in patients with moderate to severe ulcerative colitis who had previously responded to induction therapy. At week 52, 42% of patients receiving 15 mg and 52% receiving 30 mg of upadacitinib achieved clinical remission, compared to only 12% in the placebo group [11].

While anti-JAK medications offer substantial benefits in managing chronic inflammatory diseases, their use is accompanied by a range of potential side effects that require monitoring and consideration [12].

Due to their immunosuppressive properties, JAK inhibitors are associated with infections of variable seriousness. Patients receiving these medications may be more susceptible to opportunistic infections, including tuberculosis and viral infections (including herpes zoster, influenza and cytomegalovirus). For example, in a recent meta-analysis assessing the infection risk in patients on a systemic JAK inhibitor for a dermatologic indication, no difference in the rate of severe infection was detected between patients treated with medication and patients receiving placebo. On the contrary, patients receiving JAK inhibitors show higher rates of upper respiratory tract infections, Varicella Zoster and Herpes Simplex Virus infections [13].

Cardiovascular events are another possible risk associated with JAK inhibitors [14]. Some studies have indicated a higher incidence of adverse cardiovascular events, including heart attacks and strokes, venous thromboembolism and pulmonary embolism. This has raised questions about the long-term cardiovascular safety of these agents and the need for careful patient selection and monitoring. In a deeper analysis of the cardiovascular events registered in phase II and III clinical trials, cardiovascular events were only reported in patients with preexisting risk factors or patients receiving the induction dose of tofacitinib [15]. Furthermore, according to a recent meta-analysis, the risk of major cardiovascular events was not significantly different between patients receiving JAK inhibitors and patients receiving anti-TNF-α agents [16]. However, determining lipid profile and assessing cardiovascular risk is recommended before therapy initiation [17].

Other common side effects experienced by patients on JAK inhibitors for IBD or other affections include upper respiratory tract infections, acne, nausea, headaches, and elevated liver enzymes. These side effects are generally mild to moderate but can impact adherence to therapy if not managed appropriately. Musculoskeletal issues such as joint pain or muscle aches have also been reported [12].

Although head-to-head trials comparing the effectiveness and safety of JAK inhibitors are unavailable, comparative data can be retrieved for retrospective analyses or meta-analyses. In UC maintenance trials against placebo, tofacitinib and upadacitinib both achieved 3.92 RR in obtaining clinical remission, whilst filgotinib achieved an RR of 2.52; tofacitinib showed an RR of 3.16 in determining endoscopic response, with upadacitinib achieving a RR of 3.75 [18]. With regard to safety, according to a recent meta-analysis by Yang et al., tofacitinib 5 mg BID (OR = 0.7) and filgotinib 200 mg QD (OR = 0.592), followed by Upadacitinib 15 mg QD (OR = 0.556) were most frequently associated with major cardiovascular events. Such findings show that adverse cardiovascular events mainly occur in the maintenance phase and after prolonged treatment periods. Furthermore, the occurrence of patients receiving 15 mg as a maintenance dose could be justified by using the lower dosing in patients with preexisting cardiovascular risk factors [19]. Finally, UC patients on filgotinib (RR = 1.27) and tofacitinib (RR = 1.42) and CD patients on Upadacitinib (RR = 1.57) showed elevated risks of any infections [20].

### 1.3. JAK Inhibitors and Cancer

Another concern associated with JAK inhibitors is the risk of malignancy. Clinical studies have suggested a potential increase in the incidence of certain cancers, such as lung cancer and lymphoma, particularly in older patients or those with additional risk factors [21]. A 2020 meta-analysis included clinical trials and cohort studies involving adult patients with IBD, rheumatoid arthritis, ankylosing spondylitis, or psoriasis. The incidence of NMSC (non-melanoma skin cancer) was 0.51 per 100 patient-years (compared to 0.27 per 100 patient-years for patients exposed to comparator); similarly, the rate of other malignancies was 0.75 per 100 patient-years (compared to 0.18 per 100 patient-years for patients exposed to comparator) [22]. A broad 2022 meta-analysis encompassed phase II/III/IV trials and long-term extension studies of JAK inhibitors for several indications (rheumatoid arthritis, psoriatic arthritis, psoriasis, axial spondylarthritis, inflammatory bowel disease or atopic dermatitis). No significant differences in the rates of malignancies were calculated when JAK inhibitors were compared with both placebo and methotrexate. On the contrary, using JAK inhibitors resulted in a higher incidence of all malignancies than anti-TNF-α [23].

However, no clear association between JAK inhibitors and cancer has ever been pointed out. For this reason, with this narrative review, we aim to comprehensively analyse such a topic, discussing evidence deriving from randomised clinical trials, meta-analyses, retrospective studies, and real-world evidence. Table 1 summarises evidence from phase II and III clinical trials of JAK inhibitors in IBD.

### 1.4. Possible Molecular Mechanisms of Carcinogenesis Induced by JAK Inhibitors

Possible molecular mechanisms of JAK inhibitors carcinogenesis are unknown and can only be hypothesised or speculated.

JAK inhibitors can potentially contribute to carcinogenesis through several molecular mechanisms, primarily related to their broad impact on cytokine signalling and immune function. One key pathway involves the disruption of regular immune surveillance. As a downstream effect, JAK inhibitors suppress the activity of various immune cells, including natural killer (NK) cells and cytotoxic T lymphocytes (CTLs), which are critical for identifying and eliminating cancerous or pre-cancerous cells [27]. By dampening the function of these cells, JAK inhibitors may allow transformed cells to escape immune detection and proliferate unchecked, especially given the reduced immunosurveillance associated with ageing or other immunosuppressive conditions [28]. In particular, specific interferons, interleukins or TNFα can promote apoptosis in cancer cells or inhibit angiogenesis, the formation of new blood vessels that tumours need to grow. By upstream modulating these cytokine signals, JAK inhibitors could inadvertently promote a microenvironment that favours tumour growth and survival [29].

Furthermore, JAK inhibitors can directly affect signalling pathways within cells that control proliferation and survival. The JAK/STAT pathway regulates cell cycle progression, DNA repair, and apoptosis. While these drugs are designed to inhibit this pathway, they may not completely block all downstream signalling events in all cell types. Signalling, and partial inhibition of JAK/STAT could lead to aberrant signalling, resulting in uncontrolled cell growth or resistance to apoptosis [30].

Finally, some JAK inhibitors have shown selectivity for specific JAK isoforms over others. This selectivity can lead to an imbalance in cytokine signalling, potentially favouring the activation of alternative pathways that promote tumour growth or metastasis. Moreover, the off-target effects of JAK inhibitors on other kinases or signalling molecules cannot be ruled out, which could contribute to their carcinogenic potential. The specific contribution of each of these mechanisms likely varies depending on the particular JAK inhibitors, the genetic background of the individual, and other environmental factors [5].

## 2. Tofacitinib

Tofacitinib is an oral pan JAK inhibitor that inhibits JAK-1, JAK-3, and, to a lesser degree, JAK-2 and TYK-2 [31]. It is approved for many immune-mediated inflammatory diseases and emerged as a new tool in UC in 2018 [32].

Reported adverse events of tofacitinib encompass opportunistic infections such as herpes zoster and major and minor adverse cardiovascular events, including deep vein thrombosis and pulmonary embolism. Furthermore, worries about the involvement of tofacitinib in the development of malignancies have been raised [33].

Expressly, many studies have evaluated the risk of cancer following tofacitinib treatment, but there are still inconsistent findings.

The efficacy and safety of the drug were first tested in patients with moderately to severely active UC in the induction trials (OCTAVE 1 and OCTAVE2), which have reported only two cases of non-melanoma skin cancer in the 10 mg b.i.d. group. Subsequently, during an observation period of 52 weeks, maintenance trial OCTAVE SUSTAIN claimed four cases of NMSC and one of ductal breast carcinoma. The latter was observed in a patient who received a placebo in induction and maintenance. In a deeper analysis of the six mentioned cases of NMSC, all had previous exposure to thiopurines, and four out of six had a prior history of NMSC [25]. However, a long-term assessment is crucial in detecting adverse events of special interest with long latency periods, such as malignancies. The open-label, long-term extension study OCTAVE Open trial considered tofacitinib safety in UC as the primary endpoint. The incidence rates, calculated in ≤7.0 years of observation, were 1.03 (95% CI 0.67–1.52) for malignancies different from NMSC and 0.75 (95% CI 0.45–1.19) for NMSC. Nevertheless, there were six deaths in the study, all related to malignancies different from NMSC in the tofacitinib 10 mg b.i.d. group with an incident rate of 0.33 (95% CI 0.12–0.73). The causes of death were, respectively, hepatic angiosarcoma, metastatic adenocarcinoma, cholangiocarcinoma, acute myeloid leukaemia, cardiac arrest (in the setting of malignant lung cancer), and multiple organ dysfunction syndrome (in the setting of malignant melanoma) [34].

Pooled and integrated analyses of clinical trials and open-label extension studies play a crucial role in assessing long-term safety profiles and evaluating cancer development risk factors. According to pooled analyses of NMSC incidence up to 30 months in the tofacitinib UC clinical program, a Cox regression identified a higher occurrence of NMSC in patients with prior history of NMSC, age ≥ 65 years and prior anti-TNF-α failure. In the same study, known NMSC risk factors such as immunosuppressant exposure and white race were not found to be significant risk factors in this analysis [35]. On the other hand, in a post hoc analysis of the same cohort, no differences emerged in the occurrence of malignancies between ever-smokers (current smokers or ex-smokers) and never-smokers [36]. An age-stratified analysis, including an overall cohort of 1157 patients with ≤6.8 years’ tofacitinib treatment, showed that age was a statistically significant predictor of both malignancies different from NMSC and NMSC. Furthermore, the duration of UC was a predictor of malignancies different from NMSC; the history of NMSC and prior anti-TNFα failure were predictors of NMSC [37].

**Figure 3:**
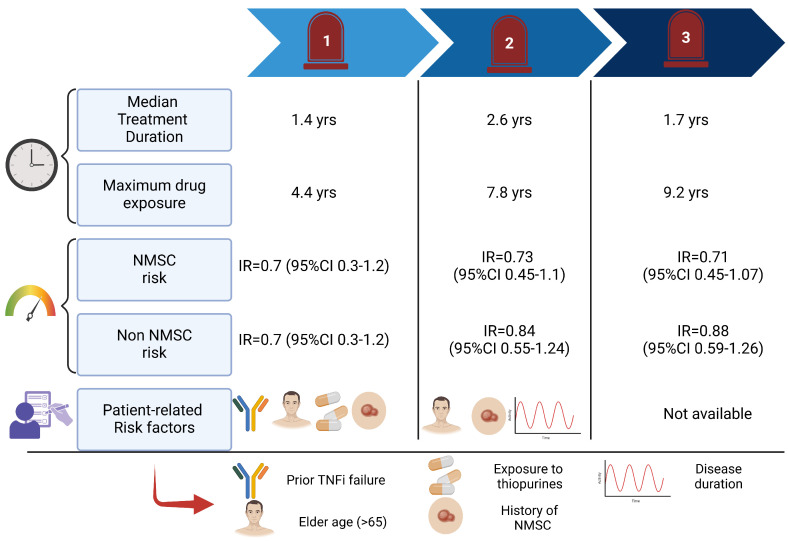
Long-term tofacitinib safety. The results from three integrated analyses, based on data from three phase 2 and phase 3 induction studies, a phase 3 maintenance study, a long-term extension study, an open-label, and a phase 3b/4 study. NMSC: non-melanoma skin cancer; IR: incidence rate; CI: confidence interval; TNFi: tumour necrosis factor inhibitors. REFERENCES: milestone 1: [38]; milestone 2: [39]; milestone 3: [40].

Three integrated analyses have been published based on the patients enrolled in phase III trials and open-label extension (Figure 3). In 2019, Sandborn and colleagues performed an integrated analysis accounting patients from phase 2, phase 3 and open-label extension studies, with a median of 1.4 years of follow-up (range 0–4.4 years, 1612.8 patient-years of exposure). In the overall cohort, 11 malignancies (excluding NMSC) were observed, with an IR of 0.7 (95% CI, 0.3–1.2). 11 cases of NMSC were also reported, with a squamous cell carcinoma/basal cell carcinoma ratio of 7:6 [38]. Data pooled from the completed global clinical developmental program up to 7.8 years of tofacitinib exposure in UC were still encouraging. In the overall cohort, 26 patients developed cancer and the IRs for all tofacitinib doses were 0.84 (95% CI 0.55–1.24) for malignancies different from non-melanoma skin cancer and 0.73 (95% CI 0.45–1.10) for NMSC [39]. The rate of adverse events of special interest remained stable in the aggregated analysis of safety data, with follow-up extended to 9.2 years. More in-depth malignancies (excluding NMSC) were reported in 29 patients. Most of them (24) were reported in patients with a predominant dose of 10 mg b.i.d. Malignancies included one case each of acute myeloid leukaemia, Bowen’s disease, Epstein–Barr virus-associated lymphoma, essential thrombocythemia, hepatic angiosarcoma, leiomyosarcoma, oesophagal adenocarcinoma, penile dysplasia, renal cell carcinoma, vulvar cancer, prostate cancer; two cases each of cervical dysplasia, cholangiocarcinoma, diffuse large B-cell lymphoma, lung cancer, malignant melanoma; three cases of breast cancer; and five cases of colorectal cancer. 23 NMSC were reported, mainly (17) in the 10 mg b.i.d. predominant dose [40].

Data about the safety of tofacitinib also derives from real-life studies. For example, a multicenter, retrospective observational cohort study enrolling 334 patients with 375 patient-years of follow-up reported three malignancies: 1 Kaposi sarcoma in an HIV+ patient, one incidental neuroendocrine tumour of the small bowel and one multiple myeloma [7].

Safety studies comparing tofacitinib and other biological therapies in UC are not currently available, but evidence can be retrieved from rheumatology. The results from ORAL Surveillance, a non-inferiority open-label randomised trial, show an increased risk for malignancy development with tofacitinib therapy in patients with rheumatoid arthritis (RA) when compared to treatment with tumour necrosis factor inhibitors over a median follow-up of 4.0 years. Overall, the reported hazard ratio for cancers excluding NMSC was 1.48 (CI 1.00–2.19) for tofacitinib 10 mg twice daily and 1.47 (CI 1.00–2.18) for tofacitinib 5 mg twice daily. At the same time, non-inferiority was not shown for the combined tofacitinib doses compared to a TNF inhibitor. The most frequently reported malignancy excluding NMSC among tofacitinib-treated patients was lung cancer (primarily non-small cell lung cancer). Notably, the population enrolled presented pre-existing risk factors for malignancy development (patients aged ≥ 50 years and with ≥1 cardiovascular risk factor) [41].

In contrast, in a sizeable, accurate word cohort of 10,504 patients with RA receiving tofacitinib or anti-TNFα, no significant differences in malignancy development were found. However, this result may be due to the short period of observation (mean follow-up < 1 year) [42]. A following study detailed the results of the ORAL Surveillance by pointing out that the risk of malignancies excluding NMSC with tofacitinib versus anti-TNFα was consistent through month 18 (HR < 1) and diverged beyond that time (HR > 1.8). Moreover, it focused on the role of baseline risk factors, including age and history of atherosclerotic cardiovascular disease. To this purpose, a post hoc Cox regression model analysis revealed that the incidence of malignancies tended to be higher in patients receiving tofacitinib and presenting such risk factors [43]. Other subsequent post hoc analyses of ORAL Surveillance confirmed that age > 65 years and history of smoking were associated with malignancies excluding NMSC [44,45].

It must be mentioned that translating the results from the ORAL Surveillance trial to the UC clinical program (and from the rheumatology trial to IBD in general) encounters several difficulties since the populations enrolled present different baseline characteristics. Elder age or pre-existing cardiovascular risk factors are often present in patients with RA enrolled for clinical trials [46].

In conclusion, studies conducted in real-world settings have reported minimal or no malignancies among patients with UC and rheumatologic affections receiving tofacitinib. However, different baseline characteristics of the patients must be taken into consideration to stratify the risk of cancer.

## 3. Filgotinib

Filgotinib is a selective JAK1 inhibitor, and such selectivity could confer an improved safety profile. It is approved for treating chronic inflammatory diseases such as RA and UC. The European Medicines Agency (EMA) initially approved it in September 2020 for treating moderate–severe RA. Subsequently, in 2021, it received European approval for use in active moderate–severe UC. It is currently not approved for CD [47].

Like the other JAK inhibitors, the long-term safety of filgotinib, particularly concerning the risk of developing neoplasms, remains an area of great interest. Regarding UC patients, in the phase III SELECTION trial, filgotinib was evaluated in induction (*n* = 1348) and maintenance (*n* = 664) over 58 weeks in patients with moderate–severe UC, with mean age ranging from 41 + −12.9 to 44 + −14.9 between groups. Participants were randomly selected to receive filgotinib, 200 mg, 100 mg, or placebo. Non-melanoma skin malignancy (NMSC) events occurred in 3 induction study patients and one in the maintenance phase. Notably, all patients with NMSC had previously been treated with thiopurine. Malignancies were reported in three patients: one colon cancer (filgotinib 100 mg), one breast cancer (filgotinib 200 mg) in the induction study, and one malignant melanoma in the maintenance study (filgotinib 200 mg) [8]. The long-term extension of the SELECTION trial (SELECTIONLTE; NCT02914535) is ongoing, but an interim analysis of up to 4 years of follow-up has recently been published. In total, 873 patients under treatment with filgotinib 200 mg/die and 260 patients under treatment with filgotinib 100 mg/die were accounted for. Ten malignancies (excluding NMSC) per 2053 patient-year exposure were observed in the 200 mg/die group, and six malignancies per 307 patient-year exposure in the 100 mg/die group. Similarly, 12 NMSC per 2033 patient-year exposure were observed in the 200 mg/die group, and 1 NMSC per 303 patient-year exposure in the 100 mg/die group [48].

Few real-world data are available for filgotinib in UC; the analysis. Of 91 patients with a median follow-up of 42 weeks (27–50), only one patient died from biliary sepsis in the context of a disseminated neoplasm that was not known at the time of filgotinib initiation [49].

From rheumatology literature, there is a greater availability of studies on the incidence of malignancies during Filgotinib treatment, in some cases with longer observation times. However, patient populations with RA do not always overlap with those with IBD, often due to age differences and comorbidities [50]. In the phase III FINCH 1 study for AR, filgotinib was evaluated in 475 (200 mg) and 480 with 100 mg; the mean age was 52 ± 12.8 53 ± 12 years, respectively, and the follow-up time was 52 weeks. Comparison with placebo or adalimumab was performed. During the placebo-controlled period, neoplasms (excluding NMSC) were reported in five patients: 1 (0.2%), 1 (0.3%), and 3 (0.6%) who received Filgotinib 100 mg, adalimumab and placebo, respectively [51].

A longer observation time is provided in the long-term extension study FINCH4 (NCT03025308), with follow-up extended to week 156, in which patients continued to receive filgotinib 200 mg or 100 mg or filgotinib 200 mg or 100 mg de novo. A total of 1723 patients were enrolled in the study, the reported malignancies (excluding NMSC) and NMSC were comparable between treatment groups, regardless of previous exposure to filgotinib: 2.34 to 3.88%, 0.8 (0.5 to 1.2) to 1.3 (0.7 to 2.2) exposure-adjusted incidence rate (EAIR) per 100 patient-years of exposure (95% CI) for tumours excluding NMSC and 0.9 to 1.59%, 0.2 (0.1 to 0.4) to 0.5 (0.3 to 0.8) EAIR per 100 PYE (95% CI) for NMSC [52].

Post hoc analyses sub-investigated the Japanese population in the FINCH 1 (52 weeks) and FINCH 4 (48 weeks) studies: previous reports had identified elevated lymphoma and/or malignant disease risks in Japanese RA patients compared to the general population. In this subpopulation analysis, no malignancy occurred in 114 patients completing the study at week 52 [53].

Overall, the post hoc analysis of the FINCH studies (seven studies (NCT01668641, NCT01894516, NCT02889796, NCT02873936, NCT02886728, NCT02065700, and NCT03025308) over a median of 1.6 years and a maximum of 5.6 years of exposure of 3691 patients who received filgotinib for 6080.7 patient-year exposure, showed one malignancy each with filgotinib 100 mg (carcinoma of the cervix) and placebo (malignant glioma) during the placebo-controlled period. In the long-term analysis, the EAIR of all non-NMSC malignancies for filgotinib 200 and 100 mg remained stable over time. In patients receiving filgotinib 200 mg, one diffuse large B-cell lymphoma and three non-Hodgkin’s lymphomas were reported; with filgotinib 100 mg, one 1 T-cell lymphoma and one central nervous system lymphoma were reported. EAIRs for NMSCs were 0.2 (0.1–0.4) and 0.1 (0–0.5)/100 patient-year exposure for filgotinib 200 and 100 mg. The malignancy rates, excluding NMSC, with filgotinib treatment were 0.6 and 0.5/100 patient-year exposure for filgotinib 200 and 100 mg and did not increase with filgotinib exposure time [50].

Finally, the open-label extension study of Phase II Rheumatoid Arthritis Programs DARWIN 3 included 739 patients; 59.5% of them had received ≥ 4 years of study drug, with a mean age of 53 and 52 years in the filgotinib + MTX and filgotinib monotherapy groups, respectively. In this cohort, excluding NMSC, neoplasms were found in nine (EAIR 0.5) patients in the filgotinib + MTX group and five (EAIR 0.6) in the filgotinib monotherapy group. For NMSC, six (EAIR 0.3) patients were identified in the filgotinib + MTX group and one (EAIR 0.1) in the filgotinib group. Thirteen patients had treatment-emerging malignancies: four haematological (three NHL, one diffuse large B-cell lymphoma) and nine solid tumours [54].

In conclusion, although the follow-up in the mentioned studies is relatively short in both UC and RA, the incidence of malignancy during Filgotinib therapy appears to be very low. However, further data covering a longer follow-up are still needed.

## 4. Upadacitinib

Several studies have evaluated the efficacy and safety of upadacitinib for treating inflammatory bowel disease, rheumatoid arthritis, psoriatic arthritis, ankylosing spondylitis, and atopic dermatitis.

The CELEST phase II randomised, double-blind trial evaluated the efficacy and safety of Upadacitinib, and was conducted in 220 patients with moderate–severe Crohn’s disease refractory to anti-TNF-α. During the induction period (16 weeks), patients were randomly selected to receive a placebo or upadacitinib (3 mg, 6 mg, 12 mg, 24 mg twice a day, or 24 mg once daily). During the maintenance period (36 weeks), all patients were re-randomised to receive a placebo or upadacitinib (24 mg once daily, 12 mg twice daily, or 3 mg twice daily). During the induction period, one case of NMSC was reported in a patient treated with upadacitinib 24 mg twice daily with previous exposure to azathioprine. During the maintenance period, two malignancies were reported. A thymus cancer was found in a 62-year-old man. A Hodgkin’s lymphoma was found in a 29-year-old man with a first-degree family history of non-Hodgkin lymphoma who had previously been exposed to natalizumab, vedolizumab, infliximab, adalimumab, and 6-mercaptopurine. This patient was treated with upadacitinib at the dose of 6 mg twice a day during the induction period and 12 mg twice a day during the maintenance period [9].

The CELEST open-label extension study evaluated long-term treatment with extended-release formulation of upadacitinib. A 96-month, multicentre, phase II study enrolled 107 patients who had completed the 52-week CELEST study. Patients who had received immediate-release formulation of upadacitinib (3 mg, 6 mg, 12 mg twice daily, or 24 mg once daily) in the CELEST study received extended-release formulation of upadacitinib 15 mg once daily. In the CELEST study, Patients who received an immediate-release formulation of upadacitinib (12 mg or 24 mg twice daily as a rescue therapy) received an extended-release formulation of upadacitinib 30 mg once daily. Two cases of neoplasms were reported in the group of patients treated with upadacitinib 15 mg. Both instances were skin cancers, one basal cell carcinoma and one squamous cell carcinoma. No malignancies were reported in the group of patients treated with upadacitinib 30 mg daily [55].

Phase III induction (U-EXCEL and U-EXCEED) and maintenance (U-ENDURE) trials were conducted to evaluate the efficacy and safety of upadacitinib in patients with moderate to severe CS. In the U-EXCEL trial, 526 patients were randomised to receive a placebo (176 patients) or upadacitinib 45 mg (350 patients) once daily for 12 weeks. In the U-EXCEED trial, 495 patients were randomised to receive a placebo (171 patients) or upadacitinib 45 mg (324 patients) once daily for 12 weeks. In the U-ENDURE trial, 495 patients with clinical response in the induction period were randomised to receive a placebo (165 patients), upadacitinib 15 mg (169 patients), upadacitinib 30 mg (168 patients) once daily for 52 weeks. Regarding the safety profile, the risk of developing malignancies was assessed at all study phases. No malignancies were recorded in any of the study groups during the induction period. In the maintenance period (U-ENDURE), two malignancies were observed in the group that received upadacitinib 30 mg (an invasive lobular breast carcinoma and a colon cancer) and one case of metastatic ovarian carcinoma in the group that received upadacitinib 15 mg [10].

Phase III induction and maintenance trials were also conducted to evaluate the efficacy and safety of upadacitinib in patients with moderate to severe UC. In U-ACHIEVE induction (UC1), 474 patients were enrolled and randomised to receive a placebo (*n* = 155) or upadacitinib 45 mg once daily (*n* = 319) for 8 weeks. In U-ACCOMPLISH (UC2), 522 patients were enrolled and randomised to receive a placebo (*n* = 177) or upadacitinib 45 mg once daily (*n* = 345) for 8 weeks. In U-ACHIEVE maintenance (UC3), 451 patients with clinical response to upadacitinib induction treatment for 8 weeks were enrolled and randomised to receive placebo (*n* = 149), upadacitinib 30 mg (*n* = 154), and upadacitinib 15 mg (*n* = 148) for 52 weeks. No malignancies were recorded in any of the study groups during the induction period. During the maintenance period, the highest number of cases of malignancies were observed in the group of patients treated with upadacitinib 30 mg (2 NMSC, one prostate cancer and one colon cancer). One case of invasive breast cancer was recorded both in the group of patients treated with upadacitinib 15 mg and in the placebo group [11].

Before receiving approval for inflammatory bowel diseases, upadacitinib was primarily approved for several other affections, including rheumatoid arthritis, atopic dermatitis, anchylosing spondylitis and psoriatic arthritis. For this reason, data from previous RCTs, registrative studies and meta-analyses can be derived.

For example, integrated data from five randomised controlled phase III trials in 3834 patients with rheumatoid arthritis treated with upadacitinib 15 mg or 30 mg were analysed. The safety data were compared with those of adalimumab and methotrexate. Cases of malignancies, excluding NMSC, result in being comparable between the groups, although higher rates of malignancies were found in the group of patients treated with upadacitinib 30 mg. Conversely, the incidence rate of NMSC in patients treated with upadacitinib 15 mg was consistent with that of the general population, adjusted for sex and age [56].

The safety profile of upadacitinib was also evaluated in the SELECT-COMPARE study. Data from 1629 patients with rheumatoid arthritis treated with upadacitinib or adalimumab for three years were analysed. Two cases of breast cancers, three cases of lung cancers, and three cases of melanomas were found in the group of upadacitinib while two cases of lung cancers and three cases of colon cancers were found in the group of adalimumab. Most cases occurred in patients older than 50 years. Overall, rates of malignancies, excluding NMSC, with upadacitinib and adalimumab were consistent with those in the rheumatoid arthritis population, with no significant differences between the two treatment groups [57].

A 2022 study by Burmester et al. evaluated the long-term safety profile of upadacitinib 15 mg and 30 mg in 6691 patients enrolled in clinical trials for atopic dermatitis, psoriatic arthritis, rheumatoid arthritis, and ankylosing spondylitis. The safety profile of upadacitinib was compared with that of adalimumab and methotrexate in a subgroup of patients with rheumatoid arthritis and psoriatic arthritis. Cases of malignancies (excluding NMSC) were reported in all disease states, with incidence rates consistent with the underlying disease and similar between upadacitinib and comparators, with no specific clusters of malignancies observed across disease groups. In patients with atopic dermatitis treated with upadacitinib 30 mg, the incidence rates of malignancies were higher than those with upadacitinib 15 mg, with approximately half of the cases occurring within the first six months of starting therapy. Safety profile analysis in atopic dermatitis showed a higher incidence of NMSC in patients treated with upadacitinib compared to adalimumab, with slightly higher rates for upadacitinib 30 mg compared to upadacitinib 15 mg. However, non-severe disease cases did not lead to treatment discontinuation [58].

A recent meta-analysis assessed the risk of malignancies in the upadacitinib clinical trials for different rheumatological affections. No differences in malignancies (excluding NMSC were detected between patients receiving upadacitinib and patients receiving comparators, both in rheumatoid and psoriatic arthritis. On the contrary, the rates of NMSC were higher in the upadacitinib population compared to comparators (adalimumab or methotrexate). Interestingly, no differences were detected according to exposure time to Upadacitinib (less than one year vs. more than one year of treatment). Finally, the risk factors analysis revealed a role for older age (>65 years), alcohol consumption and obesity, male sex and residency in the United States [59].

In conclusion, data from various studies in patients treated with upadacitinib have highlighted good results in terms of safety profiles and the risk of developing neoplasms. However, further long-term studies in patients with inflammatory bowel diseases are necessary to establish an even more precise and representative safety profile for this group of patients.

## 5. Discussion

The introduction of JAK inhibitors, including tofacitinib, filgotinib, and upadacitinib, has revolutionised the management of IBD. These agents provide effective, orally administered options for patients with moderate-to-severe UC and CD, particularly those who fail first-line therapies. While their clinical efficacy is well-documented in inducing and maintaining remission, concerns persist about their long-term safety, especially regarding cardiovascular events, infections, and malignancies. Regarding malignancies, it has to be noted that neoplasia is a common finding regardless of the field of medicine (rheumatology and gastroenterology). This consideration underlies our work and paves the way for future investigation.

This review analysed data from clinical trials, meta-analyses, and real-world studies to assess the cancer risk associated with JAK inhibitors in IBD treatment. The findings suggest that cancer risk is primarily influenced by patient-related factors such as age, smoking history, long-standing inflammation, and prior immunosuppressant exposure rather than by JAK inhibitor therapy itself. In particular, tofacitinib trials reported incidences of non-NMSC and other malignancies, predominantly in patients with pre-existing risk factors, such as prior thiopurine use or a history of cancer. These data are also confirmed when comparing tofacitinib and anti-TNF-α medication, particularly in RA; this may occur because the JAK–STAT blockade is upstream in relation to TNF-α blockade in the long term. Data extending to 9.2 years reaffirmed that malignancy rates remained stable and comparable to background rates observed in IBD populations. Similarly, data for filgotinib and upadacitinib showed minimal cancer occurrences, with most cases linked to known predisposing factors rather than drug exposure.

Safety analyses from rheumatology studies further emphasised that older age and prior cardiovascular risks, rather than the medications themselves, are pivotal in malignancy development. In particular, it is known that age is the main factor leading to reduced tumour immunosurveillance due to the senescence of the immune compartment and reduced tumoral antigens presentation. Factors further influencing immune response, such as JAK inhibitors or other immunomodulatory drugs, could worsen this predisposition [60].

Similarly, consideration must be given to the relationship between dosing and malignancies. As discussed, in RCT, most malignancies appear in patients receiving higher doses. However, these data are often denied by retrospective studies or meta-analysis. For sure, higher doses result in increased cancer risk; however, patients often receive higher doses for a short time (the induction period). This interval is often too short for neoplasia development or diagnosis. On the contrary, carcinogenesis or neoplasia detection often happens during the long-term maintenance phase [61].

Other environmental risk factors play a role. For example, smoking is an established risk factor for various cancers detected in JAK inhibitor trials, including lung cancer and bladder cancer. Similarly, alcohol plays a role in the development of liver, breast, and colorectal cancer. Both these agents converge with JAK inhibitors by creating a permissive environment for cancer development. JAK inhibitors suppress immune cell activity, potentially enabling transformed cells to evade immune detection. Concurrently, smoking and alcohol can cause DNA damage and genomic instability, increasing the likelihood of malignant transformation [62].

Considering the evidence and data, proper patient selection and pre-therapy cancer risk stratification are clinical practice’s most important take-home messages. An accurate and detailed assessment of patients’ risk factors is paramount to increase JAK inhibitors’ safety, thus unleashing their anti-inflammatory potential [46].

Table 2 is a proposed tool for clinical practice in which we outline the risk factors for cancer that should be considered before initiating anti-JAK therapy. It is important to note that the evidence supporting these risk factors remains limited and, in some cases, inconsistent. Consequently, none of the factors listed should be regarded as an absolute contraindication to such therapy. On the contrary, patients without such risk factors can be considered at low risk for cancer development due to anti-Jak therapy.

Finally, despite reassuring findings, continuous post-marketing surveillance and real-world evidence remain critical to clarify long-term safety profiles further, mainly as JAK inhibitors are increasingly prescribed in broader patient populations. Ongoing studies are expected to refine the understanding of cancer risks and other adverse events, enabling clinicians to balance efficacy and safety in clinical practice effectively.

## 6. Conclusions

In conclusion, JAK inhibitors represent a significant advancement in IBD management, offering effective therapeutic options for patients unresponsive to traditional therapies. Current evidence suggests that the risk of malignancies with JAK inhibitors is comparable to other immunosuppressive treatments, mainly when appropriate patient selection and monitoring protocols are implemented. Further long-term data and head-to-head comparisons with biologic agents will enhance confidence in their safety, ensuring their optimal integration into personalised IBD care strategies.

## Figures and Tables

**Table 1:** Reports of malignancies in IBD trials of Jak inhibitors.

Study	Drug	Disease	Study Design	N	Follow Up	Main Findings	Ref.
NCT00787202	Tofacitinib	UC	phase 2, multicentre double-blind, placebo-controlled	195	8 weeks	No malignancies	[24]
OCTAVE Induction 1 and 2	Tofacitinib	UC	phase 3, multicentre, randomised, double blind, placebo-controlled trial	1161	8 weeks	2 NMSC (all previously exposed to thiopurines)	[25]
OCTAVE Sustain	Tofacitinib	UC	phase 3, multicentre, randomised, double blind, placebo-controlled trial	593	52 weeks	4 cases of NMSC 1 case of ductal breast carcinoma (in a patient receiving placebo)	[25]
OCTAVE Open	Tofacitinib	UC	phase 3, multicentre, open-label, long-term extension study	944	7 years	22 NMSC: 13 basal cell carcinoma, nine squamous cell carcinoma 25 non-NMSC malignancies (4 CRC and two breast cancers	[25]
SELECTION- Induction A and B	Filgotinib	UC	phase 2b/3, multicentre, double-blind, randomised, placebo-controlled trial	1348	11 weeks	3 NMSC (all previously exposed to thiopurines) 2 non-NMSC: 1 CRC and one breast cancer	[8]
SELECTION Maintenance	Filgotinib	UC	phase 2b/3, multicentre, double-blind, randomised, placebo-controlled trial	664	58 weeks	1 NMSC (previously exposed to thiopurines) 1 melanoma	[8]
U-ACHIEVE Phase 2	Upadacitinib	UC	phase 2b, multicenter, randomised, double-blind, placebo-controlled trial,	250	8 weeks	No malignancies	[26]
U-ACHIEVE Induction	Upadacitinib	UC	phase 3, multicentre, randomised, double blind, placebo-controlled trial	474	8 weeks	No malignancies	[11]
U-ACCOMPLISH	Upadacitinib	UC	phase 3, multicentre, randomised, double blind, placebo-controlled trial	522	8 weeks	No malignancies	[11]
U-ACHIEVE Maintenance	Upadacitinib	UC	phase 3, multicentre, randomised, double blind, placebo-controlled trial	451	52 weeks	1 breast cancer (placebo) 1 breast cancer (15 mg) 1 prostate cancer and 1 CRC (30 mg)	[11]
CELEST	Upadacitinib	CD	phase 2, multicentre double-blind, randomised, placebo-controlled trial	220	36 weeks	1 NMSC (previously exposed to thiopurines) in the induction phase 1 thymus neoplasia and 1 Hodgkin lymphoma in the maintenance phase	[9]
U-EXCEED	Upadacitinib	CD	phase 3, multicentre, randomised, double blind, placebo-controlled trial	495	12 weeks	No malignancies	[10]
U-EXCEL	Upadacitinib	CD	phase 3, multicentre, randomised, double blind, placebo-controlled trial	526	12 weeks	No malignancies	[10]
U-ENDURE	Upadacitinib	CD	phase 3, multicentre, randomised, double blind, placebo-controlled trial	502	52 weeks	1 ovarian cancer (15 mg dosing) 1 CRC and one lobular breast cancer (30 mg dosing)	[10]

Data from phase II and phase III randomised controlled trials evaluating JAK inhibitors approved for clinical use in inflammatory bowel disease. Trials investigating JAK inhibitors not approved for clinical practice in specific indications were excluded. Abbreviations: UC: ulcerative colitis; CD: Crohn’s disease; NMSC: non-melanoma skin cancer; CRC: colorectal cancer.

**Table 2:** Possible risk factors for cancer development under anti-JAK therapy.

Risk Factors for Cancer	Reference
Previous exposure to thiopurines	[26,35,38]
History of NMSC	[26,35,37,38,39]
Age > 65 years	[35,37,38,39,60]
Prior anti-TNFα failure	[35,37,38]
Long-standing disease	[37,39]
Atherosclerosis and cardiovascular disease	[43]
Alcohol, obesity	[60]
